# Characteristics, risk factors, prevention and nursing measures of medical device-related pressure injuries in critically ill patients: a scoping review

**DOI:** 10.3389/fpubh.2026.1896234

**Published:** 2026-08-28

**Authors:** Liuqin Xu, Fangjin Lei, Rui Wang, Xiaojuan Ye, Jinghong Gao, Fengling Yang, Qingping Huang

**Affiliations:** 1 Department of Nursing, The Sixth Affiliated Hospital of Guangdong Pharmaceutical University, Houjie Hospital of Dongguan, Dongguan, Guangdong, China; 2 Department of Critical Care Medicine, The Sixth Affiliated Hospital of Guangdong Pharmaceutical University, Houjie Hospital of Dongguan, Dongguan, Guangdong, China; 3 Medical Department, The First Affiliated Hospital of Zhengzhou University, Zhengzhou, Henan, China

**Keywords:** characteristic, critically ill patient, medical device-related pressure injuries, nursing, prevention, risk factor

## Abstract

**Background:**

Studies on medical device-related pressure injuries (MDRPIs) in critically ill patients still face some nonnegligible challenges, impeding the development and implementation of evidence-based interventions.

**Objectives:**

The purpose of this review is to investigate MDRPIs in critically ill patients, from the aspects of characteristics, risk factors, prevention and nursing measures, to improve high-quality assessment, treatment, and management of critically ill patients with MDRPIs.

**Methods:**

A systematic literature search was conducted across major electronic databases to identify relevant studies. In addition, the websites and materials from relevant associations, professional organizations, and official departments were also searched for further information. Necessary manual search for references of the retrieved articles was performed to capture potential eligible studies. Two reviewers independently conducted study selection based on the inclusion and exclusion criteria, and then data extraction was carried out by using a pre-designed form. A comprehensive summary of the findings from the final included studies in relation to MDRPIs in critically ill patients was achieved through narrative data synthesis.

**Results:**

Eighteen articles were finally included, which were conducted in Brazil, Australia, New Zealand, Turkey, Norway, Jordan, and China, respectively, covering 15,341 critically ill patients. Various medical devices are responsible for MDRPIs in critically ill patients, mainly including endotracheal/orotracheal/nasotracheal intubation tubes. The most commonly affected anatomical locations involving the nose/nasal mucosa, mouth, lips, and ear. The MDRPIs in critically ill patients are caused by the interaction of multiple factors, which can be generally classified into three categories: medical devices, patient-specific factors, and treatment- and care-related factors. Correspondingly, the synthesized preventative and nursing measures were also categorized into the abovementioned three categories.

**Conclusion:**

MDRPIs exhibit device-dependent injury characteristics, with risk factors involving multi-dimensional interactions among medical devices, patients, and treatment and care procedures. Risk assessment, device optimization, positioning management, and skin care are core interventions for the occurrence of MDRPIs in critically ill patients. In order to provide safe and effective treatment and care services for critically ill patients, the prevention and control of MDRPIs should be guided by existing evidence, integrate innovative technologies and practical wisdom, improve the professional competence of medical staff, and enhance multidisciplinary collaboration.

## Introduction

With advances in technology and healthcare, medical devices are increasingly being used in clinical diagnosis, treatment, and care, and have become an indispensable part of modern healthcare. While medical devices play a vital role in assisting diagnosis and treatment, supporting physiological functions, and improving patient outcomes, their frequent use has also brought new health challenges, especially medical device-related pressure injuries (MDRPIs), which are increasingly regarded as major issues of healthcare quality and patient safety worldwide (1, 2). In 2016, the National Pressure Ulcer Advisory Panel defined MDRPIs as localized damage to patients’ skin and/or subcutaneous tissue (including mucous membranes) caused by sustained pressure from medical devices used for diagnostic or therapeutic purposes, that conform to the shape of the devices (3, 4). MDRPIs not only have adverse effects on patients’ body (pain) and mental health (anxiety), but may also lead to deterioration of patients’ health, secondary nosocomial infection, prolonged hospitalization and recovery time, poor prognosis, decreased quality of life, and life-threatening complications such as sepsis, tissue necrosis, and gangrene, imposing a considerable economic burden and care costs on patients and healthcare system (5–8). It is estimated that MDRPIs account for more than one-third of hospital-acquired pressure injuries, making it the third most costly disease after cancer and cardiovascular disease, and increasing wound care costs by an average of $10,708 per patient (3, 9–11). In the UK, MDRPIs typically extend hospital stays by 4 to 6 days (12). In the US, the annual cost of MDRPIs is US$26.8 billion, while the number in Australia is A$9.11 billion (11, 12).

Critically ill patients often suffer from severe respiratory, cardiovascular, or neurological conditions, requiring extensive use of medical devices for treatment, monitoring, life support, or rehabilitation, which inevitably leads to MDRPIs. In addition, compared with patients in general wards, critically ill patients have several associated factors that may increase the risk of MDRPIs, including organ dysfunction, malnutrition, simultaneous use of multiple medical devices, prolonged fixation of devices to ensure proper function, inability to reposition medical devices, difficulty in control temperature and humidity at the interface between devices and skin, and physical limitations, impaired senses, confusion, capillary fragility, and edema due to the use of vasoactive drugs, sedatives, or muscle relaxants (13–15). Studies indicated that 3 to 24% of patients in intensive care units (ICU) developed MDRPIs, and compared to ordinary patients, critically ill patients used medical devices were 2.4 times more likely to develop MDRPIs (16, 17). An Australian study showed that the overall incidence of MDRPIs was 27.9%, with 68% occurring in ICU patients (15). Another study reported that 72.2% of pressure injuries in critically ill patients were caused by medical devices (18).

MDRPIs is increasingly regarded as a key indicator for assessing patient safety and quality of care in healthcare facilities. In general, MDRPIs are preventable, however, their high incidence necessitates appropriate interventions to reduce the adverse impact on patient outcomes and healthcare costs. As the most direct caregivers for critically ill patients, nurses have continuous contact with patients and play a central and irreplaceable role in identifying, assessing, preventing, and managing MDRPIs (2, 12). Recent evidence suggested that nurses’ clinical judgment, risk awareness, and timely interventions form the basis of strategies for preventing MDRPIs, which effectively reducing MDRPIs prevalence and protecting patients from secondary harm (12, 19). For instance, nurses’ knowledge level, clinical competence, and systematic skin assessment skills are significantly associated with a reduction in the incidence of MDRPIs (2). Nurses who are skilled at identifying early risks and consistently apply evidence-based practices are more effective in preventing the development of injuries.

To date, despite growing clinical awareness and research interest, research on MDRPIs in critically ill patients still face many limitations and knowledge gaps. First, the evidence in relation to characteristics and pathogenesis of MDRPIs in critically ill patients remains largely inconsistent and fragmented, with differences in study settings, target population, sample size, methodological tools, and regional representativeness, hindering a comprehensive understanding (12). Second, the process and nature of MDRPIs experienced by this unique group of critically ill patients is unclear, and the preventative and nursing measures tailored to this group are not clearly articulated in the literature (20, 21). Third, although the Clinical Practice Guideline has incorporated content related to MDRPIs, clinical practice for critically ill patients in different medical settings still faces challenges such as insufficient knowledge, inconsistent guideline implementation, non-standard interventions, and insufficient attention from nurses (6, 22). The aforementioned deficiencies limit the development and implementation of optimal, evidence-based coping strategy for the prevention and management of MDRPIs, which is a key component of high-quality care for critically ill patients (12, 23). Thus, the purpose of this study is to conduct a scoping review of the literature on MDRPIs in critically ill patients to obtain a comprehensive account of the current state of knowledge and evidence relative to characteristics, risk factors, preventive and nursing measures. The findings may play a role in providing uptodate evidence for targeted clinical protocols and interventions, and improving clinical practices and medical policies for critically ill patients, thereby effectively preventing and reducing the occurrence of MDRPIs.

## Methods

### Study design

Scoping review is intended to synthesize the uptodate literature and determine existing knowledge gaps and directions for further studies (24). Given the absence of reviews on prevention and nursing measures for MDRPIs in critically ill patients, we adopted a scoping review to map the breadth and depth of research in this field, which enables us to identify current challenges, discuss potential implications for medical service quality-related policies, and provide future directions for researchers. This review was conducted according to the guidance of the Preferred Reporting Items for Systematic Reviews and Meta-Analyses Extension for Scoping Reviews (PRISMA-ScR) statement (25). In order to map the current literature describing MDRPIs in critically ill patients, the PCC strategy (population, concept, context) was used to elaborate the research question, defining P: critically ill patients, C: characteristics, risk factors, prevention and nursing measures of MDRPIs, and C: use of medical device (26). Thus, the following research questions were mainly addressed:

(1) What are the characteristics of MDRPIs in critically ill patients?
(2) What are the risk factors for the occurrence and development of MDRPIs in critically ill patients?
(3) What clinical and nursing measures are adopted to prevent MDRPIs in critically ill patients?
(4) What are the key nursing elements for the critically ill patients occurred MDRPIs?
(5) What are the challenges and future research directions in existing studies and clinical practices concerning MDRPIs in critically ill patients?

### Search strategy

A literature search, using Medical Subject Headings (MeSH) from the US National Library of Medicine and free-text keywords, was conducted in electronic databases PubMed, Web of Science, Embase, and CINAHL EBSCO, spanning from their inception to December 31, 2025. Meanwhile, the websites and materials from relevant academic associations, professional organizations, and official departments were also searched for further information. Key search terms included patient-related terms (e.g., “critically ill,” “critical illness,” “ICU,” “intensive care,” “critical care,” “long term care,” “life support care”), medical device-related terms (e.g., “medical device,” “device-related,” “equipment,” “supplies”), pressure-related concepts (e.g., “pressure injury,” “pressure ulcer,” “pressure sore,” “pressure damage,” “medical device related pressure injury,” “MDRPI,” “decubitus,” “bedsore,” “bed sore,” “stress ulcer,” “stress injury,” “bed ulcer,” “skin injury”), and nursing-related terms (e.g., “nurse,” “care,” “nursing,” “nursing intervention”). These terms were tailored to each database’s specific indexing system to facilitate a comprehensive search. Only English-language articles were included in the initial search. In addition, references of all retrieved materials and relevant review articles were manually examined to avoid missing other eligible studies.

### Inclusion and exclusion criteria

The inclusion criteria are delineated based on the abovementioned PCC strategy (population, concept, context) (26, 27), which requires all components of the research question’s structure to be clearly defined: (1) P: critically ill patients; (2) C: characteristics, risk factors, prevention and nursing measures of interest; (3) C: occurrence of MDRPIs. All study types and methods, including peer-review original articles, review, experience reports, qualitative studies, case reports, gray literature, specialist consensus or opinion, editorials, and book chapters were considered. Meanwhile, given statistical power and strength of evidence, peer-reviewed studies documented the quantitative comparison of exposure factors or intervention measures were received special attention. The exclusion criteria are as follows: (1) the patients already had MDRPI prior to being critically ill; (2) animal experiments; (3) with no full-text available; (4) duplicate publications in the databases; (5) works did not answer the questions of this review. When multiple publications arose from the same research, we prioritized information from the latest study for data extraction.

### Study selection

The primary literature search results were exported into EndNote software (Version 20, Clarivate Analytics), which was used to review all citations and remove duplications. Two reviewers (LQX & JHG) independently conducted a two-stage manual screening process in duplicate. First, the titles and abstracts of all identified records were screened based on the predefined inclusion and exclusion criteria. Second, for those studies that meet the eligibility criteria or that cannot yet be fully excluded, full texts were retrieved and assessed for final inclusion. The screen results of both reviewers were compared, disagreements at either stage were resolved through discussion or consultation. A third reviewer (FJL) adjudicated the disagreements when consensus could not be achieved. The process and result of study selection were documented and reported through a narrative and a flowchart following the PRISMA-ScR statement.

### Data extraction

In order to assure the validity and quality of extracted information, two authors (FLY & JHG) independently extracted data from the final eligible articles by using a pre-designed form. The extracted data was cross-checked and disagreements were resolved through discussion or consultation with a third reviewer (QPH). The items for data extraction mainly included first author, publication date, location, patients, study design, methods, related medical devices, risk factors, prevention and nursing measures, and main findings.

### Data synthesis

Due to significant heterogeneity in assumptions, study type, research subject, reporting methods and results, cross-study aggregate estimates (e.g., meta-analysis) were precluded. Thus, data synthesis commenced with a thorough narrative summary of the key findings from the final included studies. The evidence in relation to the characteristics, risk factors, prevention and nursing measures of MDRPIs in critically ill patients were grouped and described in categories.

## Results

### Study selection and characteristics

**Figure 1:**
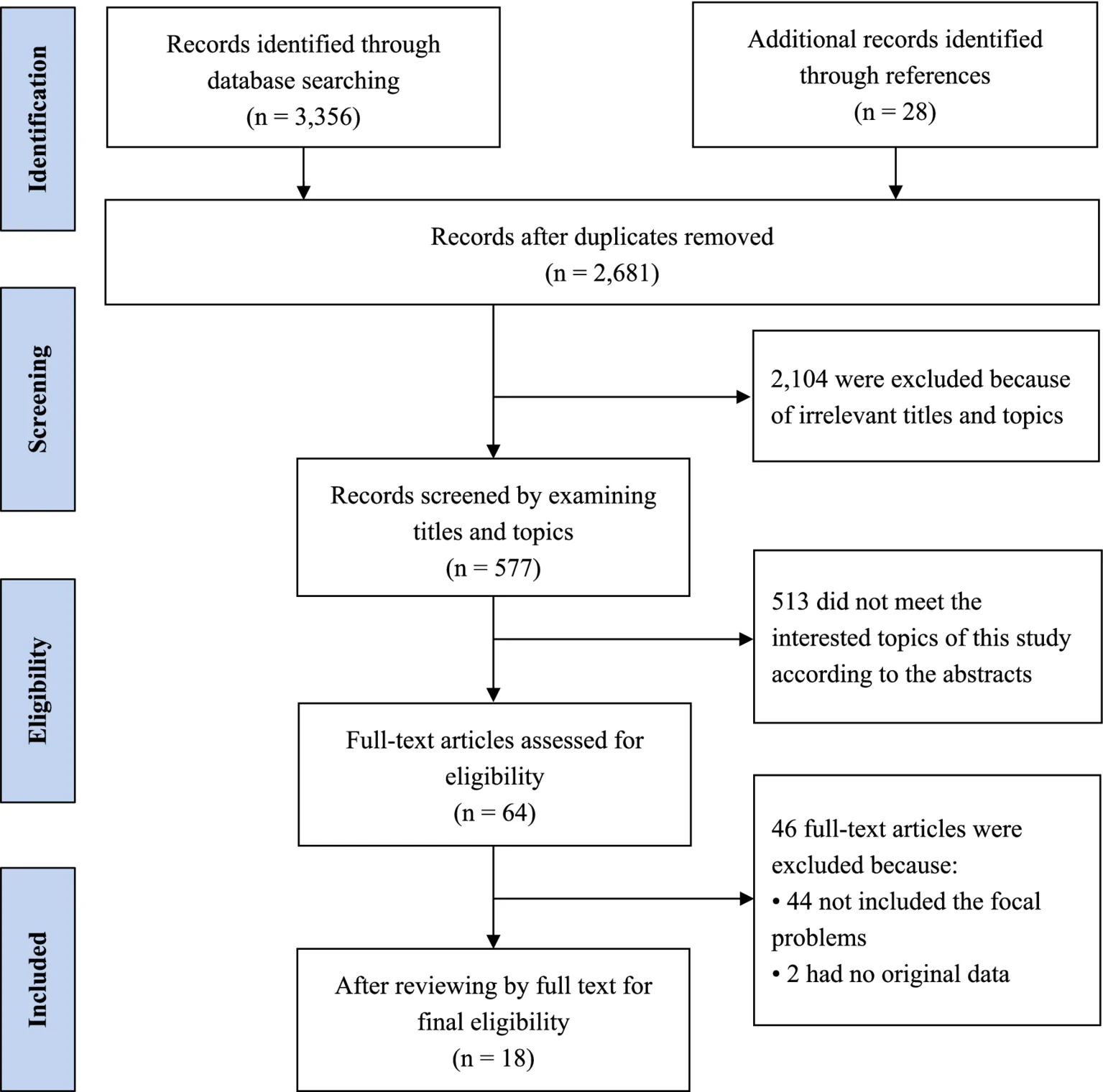
Flow diagram of the study selection process.

A total of 3,356 studies were identified through database searches, 28 additional articles were retrieved by scanning references and relevant websites, with 2,681 studies remaining after the removal of duplicates. The titles and abstracts of the preliminary identified records were screened for relevance, and 577 studies were selected for further evaluation. According to the inclusion criteria and topics of this review, 513 studies that did not involve the characteristics, risk factors, prevention and nursing measures of MDRPIs were excluded. Then, 64 studies were included for a full-text review and 18 articles were finally retained. The study selection process is presented in Figure 1.

The characteristics of the included studies are summarized in Table 1. Among the 18 studies, a total of 15,341 critically ill patients were included, with a sample size ranging from 93 to 10,084, and there were 14,409 (93.92%) from intensive care units (ICU). The included studies were conducted in seven countries: Brazil (*n* = 2) (9, 13), Australia and New Zealand (*n* = 1) (28), Australia (*n* = 3) (5, 29, 30), Turkey (*n* = 8) (8, 10, 14, 16, 31–34), Norway (*n* = 1) (35), Jordan (*n* = 1) (21), and China (*n* = 2) (6, 36), which were published from 2021 to 2025. Methodologically, the 18 included studies encompassed a majority of cross-sectional designs (*n* = 8), and 5 studies employed prospective and descriptive designs, while cohort, case–control, review, and multi-centre prospective designs was used in others 5 studies, respectively.

**Table 1:** Basic information of the final included studies (*N* = 18).

No.	First author	Year	Country	Patients (sample)	Study design	Methods	Main findings
S1	Galetto	2021	Brazil	ICU patients over 18 years (sample size *n* = 93)	Cross-sectional study	Poisson regression	Factors associated with MDRPIs were severe edema, low Braden and Glasgow scores, length of stay in ICU and hospitalization diagnosis classified as other causes.
S2	Coyer	2021	Australia, New Zealand	ICU patients aged more than 16 years (*n* = 624)	Cross-sectional point prevalence study	Logistic regression analysis	Heavier body weight, higher APACHE II scores at admission, presence of mechanical ventilation, respiratory conditions, and transfer from another ICU were factors associated with MDRPIs.
S3	de Assis	2021	Brazil	Adult patients in ICU (*n* = 125)	Cross-sectional study	Logistic regression analysis	Use of a nasal catheter, cord for orotracheal tube fixation, oximeter, intra-abdominal pressure equipment, and indwelling urinary catheter were significantly associated with MDRPIs.
S4	Erbay Dallı	2022	Turkey	ICU patients over 18 years (*n* = 172)	Prospective observational cohort study	Logistic regression analysis	Age (46–64 years), history of CVDs, administration of vasopressors, length of ICU stay ≥22 days, and mechanical ventilation requirement were independent risk factors for MDRPIs.
S5	Coyer	2022	Australia	ICU patients over 18 years (*n* = 631)	Longitudinal point prevalence study	Logistic regression analysis	The characteristics associated with the MDRPIs included total number of devices, length of time in ICU, sex with the odds increasing with being male, and APACHE II score.
S6	Weber	2022	Australia	ICU patients age ranged from 20 to 90 years (*n* = 150)	Retrospective case–control study	Logistic regression analysis	Length of stay in the ICU was the only statistically significant predictor of MDRPIs.
S7	Yalçin	2023	Turkey	Adult patients in internal and surgical ICU (*n* = 200)	Cross-sectional study	Logistic regression analysis	MDRPIs developed in approximately one of three patients hospitalised in the ICU, and the length of hospital stay and mechanical ventilator support were important determining risk factors.
S8	Aydım Kudu	2023	Turkey	ICU patients over 18 years (*n* = 213)	Longitudinal descriptive and cross-sectional study	Logistic regression analysis	The number of medical devices in the patient, the length of time the medical device was worn, and nutritional status were found to significantly increase the likelihood of MDRPIs.
S9	Karacabay	2023	Turkey	Neurology and Anesthesia-Reanimation ICU patients 18 years or older (*n* = 300)	Cross-sectional study	Chi-square test, t-test	18% of the patients occurred MDRPIs, and respiratory support device, bedridden, and experiencing non-device-related pressure injuries were associated with MDRPIs.
S10	Jia	2023	China	Adult patients in ICU over 16 years (*n* = 10,084)	Review	Meta-analysis	The incidence and prevalence of MDRPIs in adult ICU were high, and the most significant risk factor for MDRPIs was using mechanical ventilation.
S11	Saleh	2023	Jordan	Adults ≥18 years in ICU (*n* = 299)	Prospective observational study	Descriptive statistics	Prevalence of MDRPIs was 5.01, 65.8% were skin injuries and 34.2% were mucosal.
S12	Temiz	2024	Turkey	ICU patients over 18 years (*n* = 187)	Cross-sectional study	Logistic regression analysis	More than one-fourth of the patients treated in ICU developed MDRPIs, and skin type, length of stay in ICU and BMI were the determining risk factors.
S13	Tezcan	2024	Turkey	ICU patients over 18 years (*n* = 138)	Prospective and descriptive study	Logistic regression analysis	MDRPIs developed more frequently in patients with longer hospitalization days, higher PaCO_2_ levels, shorter duration of oxygenated breathing with nasal cannula or mask, and lower Braden scores.
S14	Yarkıner	2024	Turkey	Neonatal ICU patients being 0–28 days (*n* = 116)	Prospective and descriptive study	Logistic regression analysis	MDRPIs were more frequently in newborn with respiratory distress, receiving sedation, using inotropic drugs, with low gestational age and mean birth weight.
S15	Flæten	2024	Norway	ICU adult patients 18 years or older (*n* = 594)	Prospective observational cohort study	Logistic regression analysis	MDRPIs occurred mainly in the face and toes. Nearly half of the ICU-acquired PIs were related to devices, with compression stockings being the most common device.
S16	Bao	2025	China	Patients in Pulmonary and Critical Care Medicine over 18 years (*n* = 932)	Multi-centre prospective study	Logistic regression analysis	Use nasal catheter, high flow oxygen therapy, non-invasive ventilation, invasive ventilation, have chronic respiratory disease, use hormone drugs, sedatives, and abnormal skin were the risk factors of MDRPIs.
S17	Fulbrook	2025	Australia	ICU patients ≥8 years (*n* = 413)	Descriptive study	Descriptive statistics	Endotracheal tube-associated pressure injuries were the most common type of MDRPIs, effective interventions would have a significant impact on overall reduction of ICU-acquired MDRPIs.
S18	Seval	2025	Turkey	PICU patients aged 1 month to 18 years (*n* = 70)	Cross-sectional study	Descriptive statistics	The incidence of MDRPIs in PICU patients is 12.8%, with stage 1 MDRPIs most commonly developing in the nasal/oral region.

### Characteristics of MDRPIs in critically ill patients

All of the included studies reported the prevalence or incidence of MDRPIs, with rates ranging from 2.4 to 62.4%. The average/median age of the subjects was between 32.94 weeks (newborns) and 72.5 years, and more than half of the patients were males. As shown in Table 2, according to the final included studies, a wide variety of medical devices are responsible for MDRPIs in critically ill patients, mainly including endotracheal/orotracheal/nasotracheal intubation tubes (e.g., tracheostomy tube, nasogastric tube, and nasal cannula), urinary catheter, face mask, oxygen saturation probe, electrocardiograph, blood pressure cuff, cervical collar, fasteners (restrictors), and pulse oximeter. MDRPIs can occur in different parts of the body, with the most commonly affected anatomical locations including the nose/nasal mucosa, mouth, lips, ear, fingers, neck, arm, urethral meatus/genito-urinary, cheek, chest, leg, tongue, and wrist/ankles (Table 3).

**Table 2:** Medical devices related to MDRPIs in the included studies.

Medical device	Frequency	Involved studies
Endotracheal/orotracheal/nasotracheal intubation tubes		
Nasal cannula	14	S2, S3, S4, S5, S8, S9, S11, S12, S13, S14, S15, S16, S17, S18
Nasogastric tube	14	S1, S2, S4, S5, S6, S7, S8, S9, S11, S12, S13, S15, S17, S18
Endotracheal tube	13	S4, S5, S6, S7, S8, S9, S10, S11, S12, S14, S15, S17, S18
Tracheostomy tube	10	S2, S5, S6, S7, S8, S9, S11, S13, S17, S18
Orogastric tube	5	S2, S4, S14, S17, S18
Intubation tube	4	S7, S13, S15, S17
Orotracheal tube	3	S1, S2, S3
Nasoenteric tube	1	S1
Urinary catheter	13	S1, S3, S4, S5, S6, S7, S8, S9, S11, S12, S15, S17, S18
Face mask	12	S4, S6, S7, S8, S9, S10, S11, S12, S13, S14, S17, S18
Oxygen saturation probe	9	S4, S5, S8, S11, S13, S14, S15, S16, S17
Compression stockings	7	S4, S5, S9, S11, S15, S17, S18
Blood pressure cuff	6	S4, S7, S9, S11, S14, S18
Fasteners (restrictors)/tape	6	S2, S4, S8, S10, S11, S12
Cervical collar	5	S2, S7, S11, S15, S18
Electrocardiograph	4	S2, S7, S8, S11
Pulse oximeter	4	S1, S3, S9, S18
Bed/cooling blanket	3	S2, S15, S17
Peripheral intravenous catheter	2	S11, S13

**Table 3:** Anatomical locations of MDRPIs in the included studies.

Anatomical location	Frequency	Involved studies
Nose/nasal mucosa	14	S1, S2, S3, S4, S5, S6, S7, S8, S9, S11, S13, S14, S17, S18
Mouth	12	S1, S2, S3, S4, S5, S6, S7, S8, S11, S14, S17, S18
Lip	12	S1, S2, S3, S4, S5, S6, S8, S9, S11, S13, S14, S17
Arm	9	S4, S7, S8, S9, S11, S14, S15, S17, S18
Ear	8	S1, S2, S4, S5, S6, S8, S15, S17
Hands/fingers	8	S1, S4, S7, S8, S9, S13, S17, S18
Neck	8	S1, S2, S7, S8, S9, S13, S15, S17
Cheek/face	8	S1, S2, S5, S9, S11, S14, S15, S17
Leg	8	S1, S7, S8, S9, S11, S14, S15, S17
Chest	5	S2, S7, S9, S11, S15
Foot/toes	5	S13, S14, S15, S17, S18
Wrist/ankles	4	S4, S8, S11, S17
Urethral meatus/genito-urinary	4	S1, S3, S4, S11
Buttock	4	S2, S15, S17, S18
Tongue	3	S2, S6, S17
Back/scapula	3	S2, S15, S17
Perineum	2	S9, S15
Genitalia	2	S11, S17

### Risk factors of MDRPIs in critically ill patients

As shown in Tables 1, 4, the occurrence and development of MDRPIs is caused by the interaction of multiple factors, which can be generally classified into three categories: external factors of medical devices, patient-specific factors, and treatment- and care-related factors. Medical device related factors such as the number of medical devices use, length of device wear time, type and location of device are the primary risk factors for MDRPIs. Patient-related factors involving the length of stay in ICU, comorbidities, skin integrity, heavier body weight, Braden score, APACHE II scores, presence of edema, age, and malnutrition can contribute to the occurrence of MDRPIs. In addition to the use of multiple devices and patients’ own conditions, MDRPIs may also result from treatment- and care-related factors, including mechanical ventilation, high flow oxygen therapy, drug use (e.g., hormone, sedatives or vasopressors), and type of ICU.

**Table 4:** Risk factors of MDRPIs identified in the included studies.

Risk factors	Frequency	Involved studies
External factors of medical devices		
Use nasal catheter	3	S3, S10, S16
Number of medical devices	6	S3, S5, S8, S10, S11, S18
Length of medical device wear time	1	S8
Orotracheal tube use	4	S3, S9, S17, S18
Oximeter, intra-abdominal pressure equipment, and indwelling urinary catheter	1	S3
Patient-specific factors		
Severe edema	2	S1, S10
Low Braden and Glasgow scores	3	S1, S13, S10
Higher APACHE II scores	3	S2, S5, S10
Length of stay in ICU	11	S1, S4, S5, S6, S7, S9, S10, S11, S12, S13, S15
Heavier body weight	2	S2, S12
Have chronic diseases	5	S2, S4, S10, S14, S16
Nutritional status	1	S8
Age/gestational age	5	S4, S10, S11, S14, S18
Skin type	2	S12, S16
Treatment- and care-related factors		
Mechanical ventilation	5	S2, S4, S7, S10, S16
High flow oxygen therapy	1	S16
Use hormone/vasoactive drugs	3	S10, S15, S16
Use sedatives	3	S14, S16, S18
Transfer from another ICU	1	S2

### Clinical and nursing interventions

Despite the complex and multifactorial pathogenesis of MDRPIs, the narrative synthesis of preventative measures from the 18 studies identified several consistent themes across device, patient, treatment, and nursing related domains. In accordance with the classification of the abovementioned risk factors, this study also summarizes the clinical and nursing interventions into aspects related to medical devices, patients, treatment, and care (Table 5). For example, in order to prevent and control MDRPIs, it is suggested that medical devices should be compatible with patients (e.g., appropriate type, size, and securement position) and repositioned at regular 3 h intervals to offload mechanical pressure (28, 37). For patients, measures including evaluate the skin underneath and around the devices to identify the signs of MDRPIs at least twice a day, and reduce or redistribute the pressure at the interface of the device with the skin, are specifically emphasized (19, 23, 28). Many treatment- and care-related measures, such as allocation of sufficient necessary resources, establishment of standardized management procedures, continuous quality improvement efforts through multidisciplinary collaboration, and development of specific risk assessment tools, are central to MDRPIs prevention and crucial for nursing practice (6, 31, 32).

**Table 5:** Clinical and nursing interventions for MDRPIs in critically ill patients.

Target objective	Clinical management measures	Nursing interventions
Medical devices	Choosing medical devices that are compatible with patients and with appropriate type, size, and securement position	Using safe device fixation techniques in line with clinical guidelines, and regular monitoring the tension of fixation
	Using soft devices to reduce pressure on the skin (e.g., soft silicone latex type nasal prong or mask)	Reducing or avoiding the traditional practice of fixing the tube with gauze or securing the tube ties tightly
	Changing the structure of medical devices or developing new devices to avoid MDRPIs	Repositioning devices at regular 3-h intervals to offload mechanical pressure (11, 28)
	Using prophylactic dressings under devices to alleviate pressure and assist with microclimate under the devices	Periodically evaluating the contact area of medical devices
	Placing silicone border masks or hydrocolloid dressings on bony prominences	Avoiding layering dressings to prevent additional pressure
	Applying barrier films to the skin underneath and surrounding the medical devices	Standardizing dressings management, replacing dressings immediately if they become damp, shifted, or soiled
	Securing devices to prevent continuous movement of the devices and friction to the skin, if possible	
	When treatment conditions permit, removing the unused or unnecessary devices as soon as possible	
Patients	Requesting patients’ regular self-assessment of comfort	Regularly rotating or repositioning the patients (6, 28, 31)
	Reducing or redistributing the pressure at the interface of the device with the skin	Conducting routine and proper skin care, do not perform incorrect skincare procedures
	With special attention to more vulnerable patients with marked weight loss, diabetes, decreased skin turgor or edema	Managing the skin’s microclimate underneath and surrounding the device (e.g., humidity and temperature)
	Supplementing with zinc or vitamin C to promote the repairment of injured skins or tissues	Continuously monitoring the integrity of device-skin interface
	Enhancing nutritional support	Routinely evaluating the skin underneath and around the devices to identify signs of MDRPIs at least twice a day (19)
Treatment and care	Establishing comprehensive and feasible standardized procedures for the assessment, prevention, reporting, and quality control of MDRPIs	Establishing MDRPIs management system, standardizing nursing framework based on official guidelines, and taking MDRPIs as an indicator to measure nursing quality
	Conducting professional continuing education and tailored training about the management of MDRPIs for clinicians	Improving nursing quality by establishing multidisciplinary team, which includes all healthcare providers, not just nurses
	Improving treatment quality through establishing multidisciplinary team, and increasing communication and collaboration among all healthcare providers	Developing specific risk assessment tools for MDRPIs, and conducting regular risk assessment for the patients, keeping dynamic monitor and report of MDRPIs
	Developing unit-specific prevention bundles of MDRPIs to standardize the timely, evidence-based measures for the critically ill patients	MDRPIs-related guides, posters, and brochures are prepared and placed in accessible locations of workplace to ensure that nurses can easily access necessary information
	Increasing the use of support surfaces and pressure-reducing interventions to prevent the MDRPIs	Conducting continuing education and in-service training for nurses on the prevention and management of MDRPIs
	Protecting the skin with dressings in high-risk areas such as the nose bridge or edges of the device	Raising awareness among nurses about the preventive protocols and practical care of MDRPIs
	For respiratory treatment, preferentially using non-invasive ventilation mask that with soft material, good elasticity and air permeability, and appropriate degree of tightness	Developing tools to regularly evaluate the nurses’ knowledge of MDRPIs care, and the quality and effectiveness of the MDRPIs-related nursing interventions
		Implementing in-house protocols for practical care guidance focused on the prevention and nursing of MDRPIs

### Nursing elements of MDRPIs

Nursing care for the critically ill patients occurred MDRPIs is a dynamic, cyclical process involving multiple dimensions. Thus, various evidence-based nursing measures are insisted to be optimized and combined to form a comprehensive MDRPIs nursing framework. This can reduce the implementation deficiencies of individual measures, ensure the consistency and precision of nursing elements, and effectively improve the quality of MDRPIs care. Specifically, the core nursing strategy is to promote wound healing and reduce the risk of serious complications such as infection by device decompression, wound management, skin care, and individualized systemic supportive treatment, based on standardized assessment. First, accurate and dynamic risk monitoring and assessment of MDRPIs are prerequisites for implementing precision care. The assessment needs to take into account the MDRPIs characteristics, skin and tissue integrity, and overall condition. Second, device decompression that cut off the source of MDRPIs was suggested the most critical measure to block the progression of damage and promote tissue repair, since continuous pressure, shear force, and friction between the device and skin are the core triggers for the progression of MDRPIs in critically ill patients. Third, according to the MDRPIs stage and wound condition, the optimal microenvironment for wound management and healing is suggested to be created through appropriate local treatment plans such as orderly debridement, infection control, and interface temperature and humidity management. Fourth, while ensuring the therapeutic function of the medical device, it is necessary to strengthen the meticulous care of the skin and surrounding tissues to prevent damage progression. Fifth, it is essential to simultaneously implement systemic supportive therapy to improve the critically ill patient’s tissue perfusion and nutritional metabolism, which can provide a fundamental support for wound healing. In addition, establishing a multidisciplinary collaboration mechanism to leverage the technical advantages of different disciplines, jointly develop and implement individualized MDRPIs nursing plans, and establishing a strict nursing quality verification mechanism that includes each nursing measure in the daily quality control checklist and is regularly checked by specialist nurses are all considered imperative measures to improve the nursing outcomes of MDRPIs in critically ill patients.

## Discussion

Critically ill patients are often exposed to various medical devices due to the actual need for life support and monitoring, such as ventilator mask, nasal cannula, monitoring electrode, pulse oximeter, vascular access device, and drainage tubes. Although these devices provide necessary support for treatment or care, the pressure, shear force, and friction they continuously exert on the skin and soft tissues, coupled with adverse changes in the microenvironment beneath the devices, make MDRPIs an increasingly prominent nursing challenge. MDRPIs not only cause additional suffering for patients, increase the risk of secondary infection, prolong recovery time and hospital stay, and increase the economic burden, but may also influence the treatment process of the primary disease, which have become one of the key indicators of clinic and nursing quality management worldwide. Compared with traditional pressure injuries at bony prominences, MDRPIs have unique clinical characteristics, pathogenesis, prevention strategies, and nursing elements. In the present study, the characteristics, risk factors, prevention and nursing measures of MDRPIs in critically ill patients were adequately investigated. According to our knowledge, this is the first time that the comprehensive account of MDRPIs in critically ill patients was explored.

### Prevalence and characteristics of MDRPIs

The incidence of MDRPIs is relatively high among critically ill patients and shows an upward trend. A recent systematic review of the incidence, prevalence, and severity of MDRPIs in ICU showed that the incidence rate ranged from 0.9 to 41.2%, and the prevalence ranged from 1.4 to 121% (38). A study in Australia indicated that the incidence of MDRPIs in hospitals was 27.9%, with 68% of those occurring in ICU, while Hanonu and Karadag reported a 40% incidence rate in five ICUs in Turkey (39, 40). Despite the similarity of the study populations, these differences in incidence rate may be attributed to variations in the medical devices and care protocols used across different institutions.

MDRPIs have relatively distinct characteristics. While all medical devices can potentially cause pressure injuries, previous studies have reported that MDRPIs are most commonly associated with respiratory equipment (e.g., nasal cannulas, non-invasive ventilation masks, endotracheal tubes), feeding tubes (e.g., nasogastric tubes, gastric tubes, intestinal tubes), urinary catheters, and neck collars (12, 20, 31, 41). MDRPIs are typically shaped to closely match the contact surface of medical devices and are commonly occurred in areas lacking fat tissue, such as the face, ears, lips, upper nose, nostrils, and cheeks (3, 4, 23, 41, 42). It has been reported that the most common anatomical site for MDRPIs was the head and face, accounting for approximately 51%, while other sites included the lower limbs, pelvic region, upper limbs, and back, with MDRPIs prevalence rates of 27%, 7.5%, 3%, and 1.5%, respectively (43). Generally, children (especially newborns), older adults, edema patients, and people with respiratory diseases, malnutrition or diabetes are at higher risk of MDRPIs (9, 17).

### Multifactorial pathogenesis of MDRPIs

This study, through a narrative synthesis of risk factors in the 18 ultimately included studies, revealed that the risk factors for MDRPIs can be categorized into three aspects: medical devices, patients, and treatment- and care-related factors (Table 4). Firstly, regarding medical device factors, design flaws, inappropriate materials, and prolonged use are key risk points. Some medical devices do not conform to ergonomic principles, resulting in uneven pressure distribution when in contact with the skin. For example, devices made of hard materials or with sharp edges may continuously compress local tissues and hinder blood circulation (18, 37). In addition, prolonged use of medical devices on the same area can also increase the risk of skin damage (23). Secondly, at the patient level, factors such as being overweight, advanced age, malnutrition, underlying diseases such as respiratory or diabetes, pre-existing pressure injuries, limited mobility, or sensory impairment are may all significantly increase the risk of MDRPIs. Specifically, older patients experience decreased skin elasticity and reduced repair capacity, malnutrition leads to reduced tissue tolerance, mobility impairment prevents patients from changing positions independently, sensory impairment delays the early detection of pressure injuries, and underlying diseases affect patients’ tolerance and recovery ability (6, 10, 18, 33). Thirdly, treatment and care factors involve procedural standardization, assessment frequency, and the effectiveness of interventions. Medical staff may cause MDRPIs if they do not follow standard procedures, such as improper selection of medical devices, excessively tight fixation of devices, improper use of tape, failure to adjust the device position regularly, neglect of skin assessment, or fix devices in areas with less fat tissue (8, 12, 14, 42). It is evident that, due to the numerous risk factors, the incidence of MDRPIs may vary among different critically ill patients. Therefore, preventive measures from multiple dimensions, such as device optimization, patient assessment, and standardized nursing procedures, should be taken to prevent and control MDRPIs.

### Comprehensive prevention and nursing of MDRPIs

The prevention and nursing measures of MDRPIs can also be systematically constructed from three dimensions: medical devices, patients, treatment and care. Based on these comprehensive interventions, the precise prevention and nursing of MDRPIs in critically ill patients can be achieved.

For medical devices, the main focus is on the standardization and safety of devices use. First, prioritize the use of ergonomically designed devices with cushioning (e.g., soft silicone, latex nose plugs or masks), ensuring that the material is soft, the edges are rounded, and that they are regularly maintained to ensure normal function (23, 31). Second, select medical devices with appropriate size and fixation devices made of soft materials according to the anatomical structure, and regularly assess the fitness between the device and skin to avoid being too tight, too loose or excessively stretched, thereby reducing shear force, friction, and pressure on the skin and mucous membranes (6, 13, 16, 28). Third, try to avoid using multiple medical devices at the same time, assess the necessity of devices as early as possible, and remove them in a timely manner when the condition permits (41, 44). Fourth, for medical devices that must be used for a long time, develop a detailed rotation plan, such as using alternative treatments, applying appropriate pressure-reducing pads to disperse pressure, or adopting alternating or resetting strategies to dynamically adjust the device position to reduce local pressure (18, 22, 45).

At the patient level, it is necessary to focus on individualized needs and actively implement precise interventions for specific clinical departments. First, routinely assess the skin and surrounding areas that have come into contact with medical devices at least twice a day to determine the wound healing status and adjust the MDRPIs management and care plan in a timely manner (22, 23). Second, strengthen humidity and temperature control, nutritional support, routine skin care and skin barrier protection for older adults, and those with chronic diseases or malnutrition (22, 46, 47). Third, for individuals with limited mobility or sensory impairment, establish a timed postural change plan, select appropriate prophylactic dressings based on the patient’s skin condition and the type of medical devices to cushion high-risk interfaces, and use pressure-reducing tools to distribute pressure and decrease shear forces (22, 28, 48).

In terms of treatment and care, the focus is on establishing and strictly adhering to standardized procedures. First, provide continuous training and support to medical staff based on knowledge assessments to ensure that devices are used in a standardized, reasonable, and appropriate manner (23). Second, implement dynamic risk assessment, especially increasing the frequency of skin examinations for high-risk patients, and using specific risk assessment scales to guide graded early warning and prevention (11, 14, 46, 48). Third, establish a dedicated interdisciplinary team—comprising physicians, nurses, nutritionists, and rehabilitation therapists—to collaboratively develop an evidence-informed, comprehensive prevention framework and individualized nursing protocols for MDRPIs in critically ill patients, and subsequently implement targeted, timely, and protocol-driven prevention and care (2, 11, 23, 47). Fourth, educate patients and their families about MDRPIs protection knowledge, and improve patients’ self-warning and protection awareness through patient education (3, 49). Fifth, increase the applications of innovative technology, such as the introduction of intelligent monitoring devices to track pressure changes of critically ill patients in real time, forming a closed-loop management of “assessment-intervention-feedback-adjustment” to effectively reduce the risk of MDRPIs (2, 13, 50).

### Existing challenges

Although MDRPIs have garnered increasing scholarly attention and research activity in recent years, studies specifically focus on critically ill patients remain limited and face several challenges. Firstly, there is a lack of multi-center data and sufficient evidence. Although research on MDRPIs in critically ill patients has gradually increased since 2020, in terms of the 18 included studies, existing research is mostly concentrated in a few countries, especially Turkey, Australia, Brazil and China. In contrast, countries from the Americas and Africa have paid relatively less attention to this topic (12, 36). Secondly, the statistical power and persuasiveness of existing studies are relatively weak. The 18 included studies predominantly employed descriptive cross-sectional designs with limited sample sizes and relied primarily on logistic regression analysis, which collectively constrain the generalizability and causal interpretability of their findings (9, 10, 14, 16). Thirdly, the target population is relatively limited. Existing studies mainly focus on adult patients aged 18 and above, while relatively few studies have explored high-risk groups for MDRPIs such as children, older adults, or pregnant women (14, 31–33, 42). Fourthly, risk assessment tools are controversial. Existing pressure injury assessment tools—including the Braden and Norton scales—are primarily designed to evaluate patient’s activity limitation and omit critical device-specific variables such as device type, contact time, and fixation method. Consequently, despite their well-established predictive validity for general hospitalized patients, they lack sensitivity to dynamic interface pressures at device–tissue contact sites and fail to reliably identify MDRPIs risk in critically ill patients (6, 44, 50, 51). It has been indicated that, due to the lack of specific assessment tools, MDRPIs are often only discovered when they have progressed to severe stages, and are also frequently unrecorded and underreported in practice and literature (44, 52).

Fifthly, there is a lack of classification standards for mucosal injuries. Mucosal injuries in MDRPIs cannot be classified according to the traditional pressure injury staging system (32, 53). Currently, there is no internationally accepted classification tool, which makes it difficult to collect and share related data globally, and corresponding nursing measures also lack high-quality evidence (10, 31, 41). Sixthly, the collaborative management and care mechanisms for MDRPIs are still immature. Although multidisciplinary collaboration is widely recommended, existing evidence is mostly observational studies or expert consensus, lacking high-quality randomized controlled trials (RCTs) to verify their actual effectiveness in the prevention of MDRPIs in critically ill patients (8, 15, 22, 54, 55). Besides, some of the existing evidence, for instance, the narrative synthesized prevention and nursing measures for MDRPIs in critically ill patients of this review, are derived primarily from observational studies, which meaning that these evidence-informed recommendations require further validation through high-quality multi-center studies, prospective cohort studies, or RCTs.

### Future work

Given the challenges faced by existing MDRPIs-related research in critically ill patients, future research can focus on and address knowledge gaps in directions include but not limited to the follows. First, conduct high-quality cohort studies, prospective interventions, or multi-center RCTs to evaluate and validate the corresponding effectiveness of different medical device designs, fixation tools, prophylactic dressings, or interventions in the prevention and care of MDRPIs in critically ill patients (12, 32). Second, it has been suggested that the vulnerable groups among critically ill patients, such as children, older adults, diabetic patients, and patients with hypoalbuminemia should be focused on, and it is crucial to explore specific prevention and care strategies for MDRPIs in these groups (14, 33, 47). Third, develop specific risk assessment tools for MDRPIs that are suitable for critically ill patients and ward nurses, enabling early warning, detection, and intervention of MDRPIs. Existing risk assessment tools for critically ill patients (e.g., the Jackson/Cubbin scale) have shortcomings such as with too many items, lack of indicators for mucosal damage, low predictive efficacy, or inconvenience in clinical application, which need to be further improved based on clinical needs (31, 41, 51). Fourth, develop specialized classification tools, care products, and interventions for mucosal injuries, and validate the predictive efficacy and clinical applicability of the newly developed tools, ultimately establishing unified and practical classification and intervention standards for mucosal pressure injuries (6, 12, 41).

Fifth, optimize multidisciplinary collaboration model, conduct regular team training, and improve medical staff’s understanding and prevention capabilities of MDRPIs in critically ill patients, then, carry out RCTs to verify the effectiveness of the model in prevention and care of MDRPIs (8, 23). Sixth, strengthen the application and clinical translation of new technologies in the prevention and care of MDRPIs in critically ill patients. For instance, based on the data from flexible sensors or infrared thermal imagers that dynamically monitor the pressure distribution at the contact interfaces of medical devices, suitable machine learning algorithms can be employed to build a specific risk prediction model for MDRPIs (6, 50). Seventh, it is imperative to develop, institutionalize, and standardize procedures, interventions, and management frameworks for MDRPIs in critically ill patients, referencing existing guidelines and incorporating identified risk factors. These frameworks, through continuous nurse training and education as well as regular assessment of their effectiveness, may significantly contribute to the prevention and control of MDRPIs (2, 56).

### Strengths and limitations

The present study has some noteworthy strengths. Firstly, this study was conducted following the PRISMA-ScR guideline to optimize the reporting within the review and reduce publication bias, which may have acceptable research quality to provide convincing evidence. Secondly, this study, through a systematic literature search, comprehensively reviewed relevant research on MDRPIs in critically ill patients, covering the characteristics, risk factors, prevention and nursing, thus painting a complete clinical picture of MDRPIs in critically ill patients. This may play a helpful role in quickly grasping the uptodate research status in this field, identifying knowledge gaps that have not been fully explored, and laying the foundation for subsequent empirical research, systematic reviews, or interventional studies. Thirdly, given critically ill patients’ MDRPIs involve multiple disciplines, this study, by integrating heterogeneous data from different research types, regions, and healthcare facilities, can provide multidimensional evidence to enhance the understanding and management of MDRPIs in critically ill patients. Fourthly, compared to systematic reviews or meta-analyses, this scoping review has a more flexible methodological framework, which does not require strict quality assessment or data synthesis, and can more comprehensively reflect the current clinical and nursing practices as well as latest trends of MDRPIs in critically ill patients, quickly respond to actual needs, and avoid missing important information due to strict inclusion and exclusion criteria. Fifthly, according to the summary of the consensus and controversies in existing evidence, this study can provide a basis for medical institutions to develop practice guidelines, devices usage standards, and training programs that used to prevent and care MDRPIs in critically ill patients, which eventually promotes the translation and application of evidence into clinical practice.

Several limitations of this study must be acknowledged. First, only studies published in English were included in this review, consequently, findings from non-English-language publications were not captured, which potentially introducing language bias. Second, although a comprehensive literature search was performed, the possibility remains that some relevant studies were not identified, and publication bias cannot be fully ruled out. Third, during the processes of literature screening, data extraction, and information summarization, authors’ subjective judgments may influence the results. Despite the fact that this study has employed independent dual review to reduce the bias, it may still be impossible to completely eliminate subjectivity, resulting in relatively weaker objectivity in the conclusions compared to systematic reviews. Fourth, the final included literature in this research were mainly cross-sectional descriptive studies, and as a scoping review, quality assessment was not conducted for the included literature, which to some extent limits the strength of evidence, reliability, and generalizability of this study’s conclusions. Lastly, the present study did not conduct quantitative data aggregation or meta-analysis, it only summarized the characteristics, risk factors, and prevention and nursing measures of MDRPIs in critically ill patients through qualitative analysis. Therefore, this study cannot answer questions such as “which preventive measures are most effective” or “the quantitative association strength of risk factors,” which may limit the explanatory power and accuracy of our conclusions. Further studies are needed to address these issues.

## Conclusion

This study systematically reviewed the characteristics, risk factors, preventive measures, and nursing elements of MDRPIs in critically ill patients, revealing the clinical complexity and current landscape of evidence-based practice. MDRPIs exhibit device-dependent injury characteristics, with risk factors involving multi-dimensional interactions among medical devices, patients, treatment and care procedures. Existing evidence suggests that risk assessment, device optimization, positioning management, and skin care are core interventions for MDRPIs in critically ill patients, however, there is a lack of high-quality empirical studies to evaluate and validate the effectiveness. Nursing practice requires dynamic adjustment of risk assessment and interventions based on changes in the patient’s condition and the use of medical devices, while taking into account individual factors, and simultaneously emphasizes multidisciplinary collaboration. Given the existing challenges and knowledge gaps in research on MDRPIs in critically ill patients, future research is suggested to focus on conducting multi-center RCTs to clarify the effectiveness of prevention and nursing measures, developing risk assessment tools for MDRPIs applicable to critically ill patients, exploring the clinical translation and application of new technologies in MDRPIs, and improving evidence-based nursing training system and multidisciplinary collaboration model. In summary, the prevention and control of MDRPIs should be guided by existing evidence, integrate innovative technologies and practical wisdom, improve the professional competence of medical staff, and develop personalized intervention strategies, based on which ultimately provide safe and effective treatment and care services for critically ill patients.
